# Characteristics, chemical compositions and biological activities of propolis from Al-Bahah, Saudi Arabia

**DOI:** 10.1038/srep41453

**Published:** 2017-02-06

**Authors:** Yasser A. Elnakady, Ahmed I. Rushdi, Raimo Franke, Nael Abutaha, Hossam Ebaid, Mohannad Baabbad, Mohamed O. M. Omar, Ahmad A. Al Ghamdi

**Affiliations:** 1 College of Sciences, Department of Zoology, King Saud University, Riyadh 11451, Saudi Arabia; 2 Chair of Green Energy Research, College of Food and Agriculture Sciences, King Saud University, P. O. Box 2460, Riyadh 11451, Saudi Arabia; 3 ETAL Consulting and Service, 2951 SE Midvale Drive, Corvallis, OR 97333, USA; 4 Department of Chemical Biology, Helmholtz Centre for Infection Research, 38124 Braunschweig, Germany; 5 Chair of Bio-product, College of Science, King Saud University, Riyadh 11451, Saudi Arabia; 6 Chair of Engineering Abdullah Baqshan for Bee Research, College of Food and Agriculture Sciences, King Saud University, Riyadh, Saudi Arabia

## Abstract

Propolis has been used to treat several diseases since ancient times, and is an important source of bioactive natural compounds and drug derivatives. These properties have kept the interest of investigators around the world, leading to the investigation of the chemical and biological properties and application of propolis. In this report, the chemical constituents that are responsible for the anticancer activities of propolis were analyzed. The propolis was sourced from Al-Baha in the southern part of the Kingdom of Saudi Arabia. Standard protocols for chemical fractionation and bioactivity-guided chemical analysis were used to identify the bio-active ethyl acetate fraction. The extraction was performed in methanol and then analyzed by gas chromatography-mass spectrometry (GC-MS). The major compounds are triterpenoids, with a relative concentration of 74.0%; steroids, with a relative concentration of 9.8%; and diterpenoids, with a relative concentration of 7.9%. The biological activity was characterized using different approaches and cell-based assays. Propolis was found to inhibit the proliferation of cancer cells in a concentration-dependent manner through apoptosis. Immunofluorescence staining with anti-α-tubulin antibodies and cell cycle analysis indicated that tubulin and/or microtubules are the cellular targets of the L-acetate fraction. This study demonstrates the importance of Saudi propolis as anti-cancer drug candidates.

More than twenty percent of the world’s population is suffering from malignant diseases. According to the American Cancer Society, 14.1 million cancer cases were diagnosed in 2012 worldwide, and more than half of these cases occurred in economically developing countries^1^. Additionally, approximately 8.2 million cancer patients around the world lost their lives in 2012. Furthermore, by 2030, the number of cancer patients is expected to increase to 21.7 million^1^. The development of an effective cancer therapy remains one of the greatest challenges for the scientific community, with little advancement in the overall cancer survival landscape during the last two decades. The administration of different therapeutic agents for cancer treatment (which also includes those from natural sources such as Taxol or vinca alkaloids) are known to produce a variety of side effects^2^,^3^,^4^,^5^. Moreover, extensive radiotherapy usually leads to other illnesses in patients, such as hematopoietic syndrome, mucositis, and other afflictions^6^,^7^,^8^,^9^. In recent years, efforts have been made to identify naturally occurring agents that could prevent cancer development without (or with minimal) side effects.

As a traditional alternative medicine approach, honeybees and/or honeybee products (e.g., venom and propolis) have been used to relieve pain and to treat inflammation since ancient times^10^. A survey of the literature indicates a recent revival of interest in exploring the medical properties of honeybee venom and propolis^11^. Different research groups around the world have reported the antibacterial, antifungal, cytostatic, wound healing, antitumor and anti-inflammatory properties of honeybee products^12^,^13^,^14^,^15^,^16^,^17^,^18^,^19^,^20^,^21^,^22^,^23^,^24^. Propolis contains more than 300 components, including phenolic aldehydes, polyphenols, amino acids, steroids, and inorganic compounds. However, the plant origin and the collection region can substantially affect the chemical composition of propolis^25^,^26^,^27^,^28^,^29^,^30^,^31^,^32^,^33^,^34^. The pharmacological potential of propolis has been very well investigated and reported in the literature, as evidenced by the large number of related reports^25^,^26^,^27^,^28^,^29^,^30^,^31^,^32^,^33^,^34^,^35^,^36^,^37^,^38^,^39^. *In vivo* studies have also been performed in which the administration of propolis to mice or humans does not seem to lead to any side effects^40^,^41^,^42^,^43^.

Propolis is a glue material that is collected by honeybees from plants and then used for sealing cracks in hives and protecting the bees from bacterial and fungal infections^44^,^45^. Ancient Egyptians, Romans and Greeks used propolis in the past as a medicine for curing some diseases^46^. The curative properties are correlated with the bio-active chemicals that are present in propolis and have sparked an interest in further explorations. An important aspect of the properties/characteristics could be related to geographical location, because the plant sources and collecting season for honeybees are different, adding another important factor to the diversity of the propolis chemical constituents and thereby adding to the biological activities^47^. Generally, the known major components of propolis are aromatic acids, flavonoids, diterpenoid acids, phenolic compounds and triterpenoids. Since honeybees visit the flowers of different plants, these plants are the major source of organic compounds in propolis during propolis formation by honeybee secretion and metabolism. The compositional breakdown of propolis is unsurprisingly 30% wax, 50% resin and vegetable balsam, 5% pollen, 10% essential and aromatic oils and 5% other substances^48^,^49^,^50^,^51^,^52^,^53^,^54^,^55^,^56^,^57^,^58^,^59^. The reported compositions mostly come from Europe and Latin America^60^,^61^,^62^,^63^,^64^,^65^,^66^,^67^,^68^,^69^,^70^,^71^,^72^,^73^,^74^, whereas there are few reports on the propolis of Saudi Arabia^47^,^75^. Saudi Arabia makes up the major part of the Arabian Peninsula and has dry climatic and physiographic conditions, except in the eastern and southern regions, where different species of flowering plants are found^75^,^76^. Honeybees produce a considerable amount of propolis in the Al-Bahah region, where many beekeepers focus only on honey production. This survey indicates a need to investigate the effects of these dry climates on the characteristics of propolis from the Arabian Peninsula.

Therefore, in this study, propolis samples were collected from the southern region of the Kingdom of Saudi Arabia to investigate their chemical compositions and anti-proliferative activities and understand the effects of regional diversity and geographical locations relative to those of reported studies from other parts of the world.

## Results

### Effects of propolis extract on the proliferation of cancer cell lines

Initially, the effects of propolis extracts were ascertained on the proliferation of four cancer cell lines that included Jurkat (T lymphocyte leukemia), HepG2 (human liver carcinoma), A549 (human lung carcinoma), and SW756 (squamous carcinoma) cell lines. Extracts from every 30 grams of propolis were obtained in two sets of 500 ml of methanol (95%). The resulting fractions were mixed and dried. This extraction yields 4.55 g of crude methanol extract (approximately 15.2% of the starting material). Two grams of the methanol crude extract were further purified using ethyl acetate and silica gel column chromatography as described in the methods section. This approach yielded 1.57 g of ethyl acetate fraction (L-acetate, L for local propolis, and acetate for ethyl acetate). The bio-activities of the two fractions were tested using an MTT assay. Table 1 shows a comparison between the anti-proliferation activities of methanol crude fractions versus that of the L-acetate fraction as measured in the MTT assay. The four cancer cell lines showed sensitivities to both fractions. However, three of the four tested cancer cell lines, namely the Jurkat, A549 and SW756 cells, exhibited great sensitivity to the ethyl acetate fraction of the propolis in comparison to its crude methanol extract. The measured IC_50_ values ranged between 1.8 and 3.2 μg/ml. By contrast, the sensitivity of HepG2 to the L-acetate fraction was lower than that measured for the methanol crude extract, and the measured IC_50_ values were 6.3 and 3 μg/ml, respectively.

### Impedance profiling of L-acetate fractions

**Figure 1:**
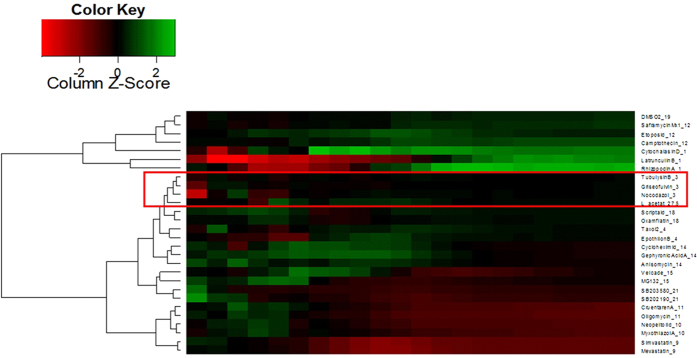
Hierarchical cluster analysis of data from impedance curves obtained with L929 cells that were incubated with L-acetate fraction and as a set of reference compounds L-acetate fraction found in close proximity to tubulysin B, griseofulvin, and nocodazol. The three compounds are known to interact with tubulin as a cellular target.

**Figure 2:**
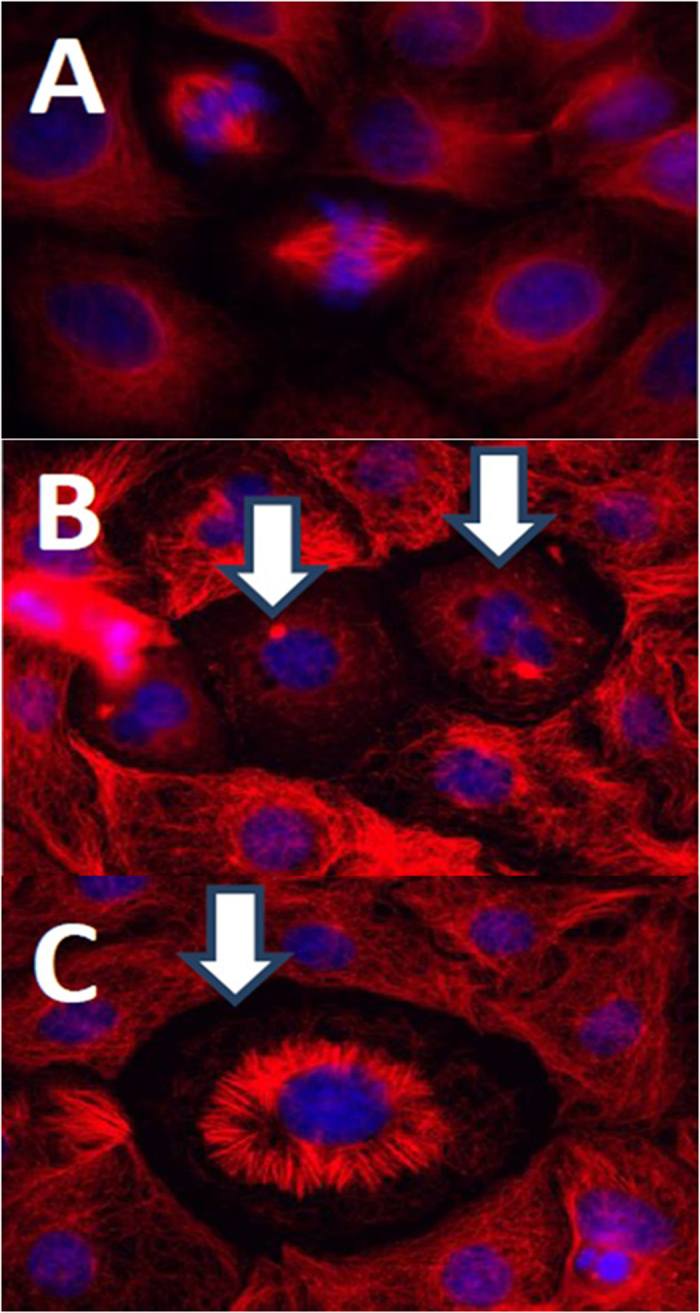
Effect of L-acetate fraction on the microtubules of PtK2 cells as shown by immunofluorescence technique using anti-α-tubulin antibodies (red). Nuclei stained with DAPI (blue). (**A**) Control Cell, (**B**), and (**C**) treated cells (100 μg/ml, 24 h).

To obtain insight into the way in which L-acetate fraction inhibit the growth of the treated cells, we employed a modern chemical biology technique to test the effects of propolis extracts on the profiles of L-929 cells (mouse fibroblasts) by monitoring the impedance of the treated cells followed by cluster analysis with compounds with known mechanisms of action. The principle of the impedance profiling approach is that compounds with a similar mode of action induce similar time-dependent impedance curves. Thus, when the curve is induced by a compound (or extract) of interest, it is compared to the curves of the reference compounds whose mode of action is known, providing hints about the action of the new compound. The impedance curves of the L-acetate fraction were compared to those of a set of 27 compounds covering a broad activity spectrum. As shown in Fig. 1, the L-acetate fraction was found to cluster with tubulysin B^77^, griseofulvin^78^, and nocodazol^79^. The three compounds are known to bind to tubulin and interfere with microtubule function. In other words, the activity of the L-acetate fraction seems to interact with tubulin as the cellular target. This finding was supported by the immunofluorescence staining of PtK2 cells using anti-α-tubulin antibodies. As shown in Fig. 2, the treatment of PtK2 cells (potoroo kidney cells) with a 100 μg/ml L-acetate fraction induces the de-polymerization of interphase microtubules as well as nuclei fragmentation (a hallmark of apoptosis). However, the de-polymerization effect of L-acetate was only observed after a long incubation time (24 hours).

**Figure 3:**
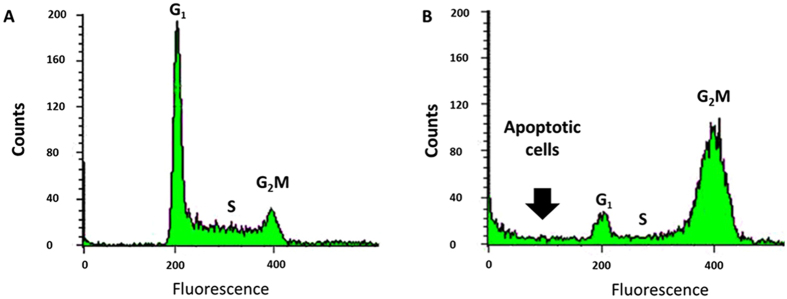
L-acetate fraction of Saudi proplis induced cell cycle arrest at G_2_M-phase in Jurkat cells. (**A**) Control cells, (**B**) Treated cells (100 μg/ml, 24 hours).

Because the compounds that bind tubulin usually caused G_2_M cell cycle arrest in the treated cells^80^, we tested the effects of the L-acetate fraction on the cell cycle distribution of Jurkat cells. As shown in Fig. 3B, Jurkat cells were accumulated over the G^2^M-phase 24 hours after treatment with 100 μg/ml of L-acetate fraction in comparison to the control cells (Fig. 3A). We could also detect a substantial increase in the number of apoptotic cells.

Consequently, we tested the effect of the L-acetate fraction on the induction of apoptosis in Jurkat cells using AnnexinV-FITC/PI staining and flow cytometry. This staining was chosen on the basis of the observation that soon after initiating apoptosis, the cells translocate their membrane phosphatidylserine (PS) from the inner face of the plasma membrane to the cell surface. Once it reaches the cell surface, the PS can be detected easily by staining it with a fluorescent conjugate of Annexin V, a protein that has a high affinity for PS. As a result of apoptosis initiation, the membrane loses its integrity, leading to the diffusion of PI (Propidium Iodide) into the cells, and it binds to the DNA. Therefore, the staining of a cell with Annexin-V-FITC indicates that the cells are in an early apoptotic stage, and staining the cells (DNA) with PI indicates that the cell is in a late apoptotic or necrotic phase.

**Figure 4:**
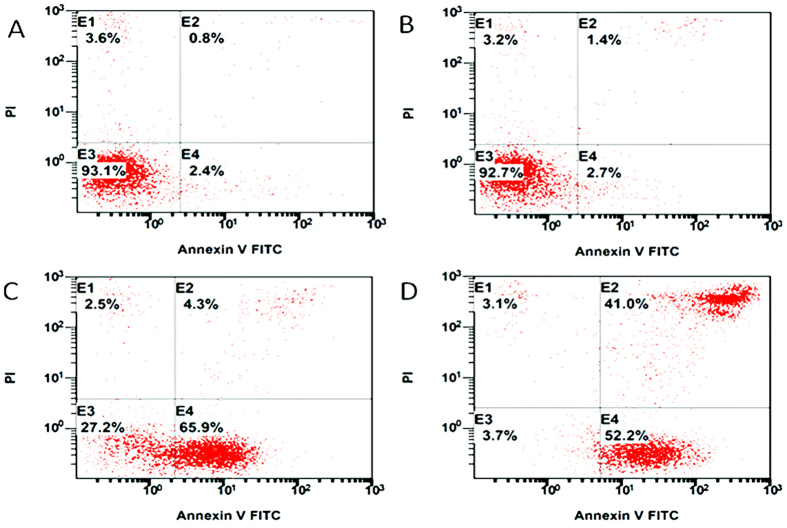
Induction of apoptosis in Jurkat cells after treatment the cells with L-acetate fraction of saudi propolis. After incubation times cells were stained with anexinV-FITC and PI and analysed with flow cytometry. (**A**) control (mehanol), (**B**) treated (100 μg/ml, 3 h), (**C**) treated, (100 μg/ml, 24 h), and (**D**) treated (100 μg/ml, 24 h).

As shown in Fig. 4, the control sample (Fig. 4A) was treated only with methanol (-ve control), and it displayed more than 93% living, non-apoptotic cells (E3). After three hours of incubation with 100 μg/ml L-acetate, the cell distribution remained almost unchanged (Fig. 4B). However, after nine hours of incubation, the percentage of living cells was dramatically decreased (from 92.7 to 27.2), and we observed a huge increase in early-stage apoptotic cells (Fig. 4C). After 24 hours of incubation, we observed only 3.7% living cells and 52.2% early stage apoptotic cells, and 41% of the cell population was in the late apoptotic stage (Fig. 4D). These results clearly indicate that the L-acetate fraction of the Saudi propolis induces apoptosis in Jurkat cells.

### Effects of the L-Acetate Fraction on Rats (*Rattus norvegicus*)

**Figure 5:**
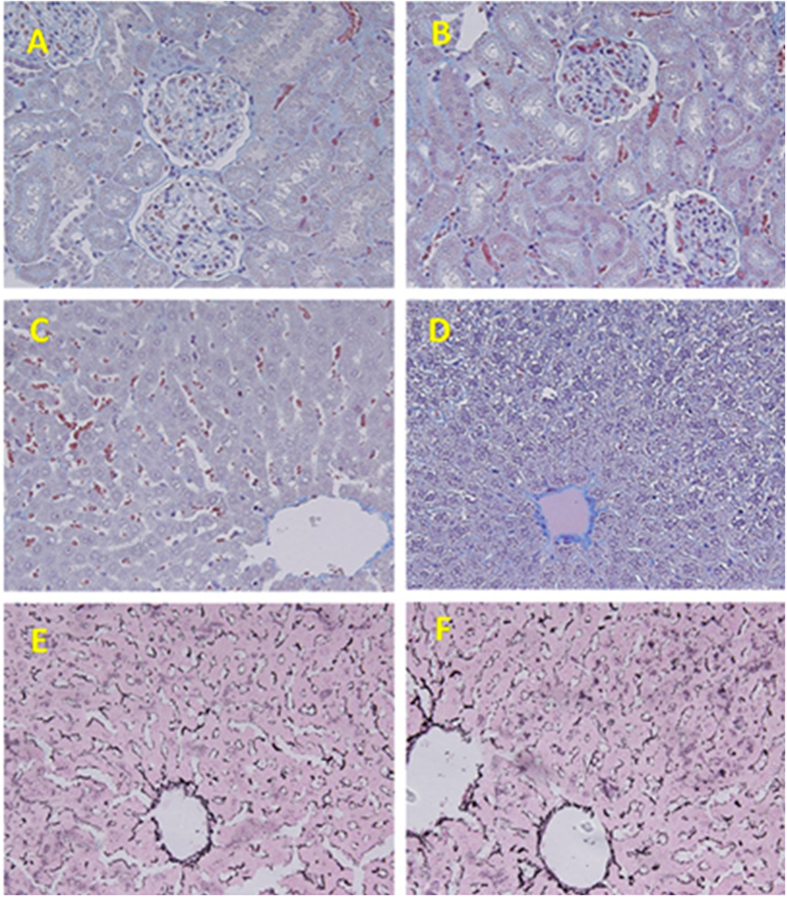
Representative photomicrograph of the histopathological examinations of both kidney and liver. (**A,B**) Control and treated renal tissues stained with Masson Trichrome (X 400). (**C,D**) Control and treated hepatic tissues stained with Masson Trichrome (X 400). (**E,F**) Control and treated hepatic tissues stained with Reticulin (X 400).

We tested the effects of the L-acetate fraction on physiological and histological functions in male Wistar rats (*Rattus norvegicus*). The rats were divided into two groups, each of which contained ten rats. Group 1 served as the control and only received the vehicle. Group 2 received a daily intraperitoneal dose of 1 mg/kg body weight of the ethyl acetate fraction. The histological architecture of the renal tissues from both the control and treated rats were shown to be normal without any significant changes in either the glomeruli or convoluted tubules (Fig. 5A and B). The control hepatic tissues showed normal histological architecture. Unlike the control, a number of vacuolated hepatocytes and abnormally narrowed hepatic sinusoids were found in the sections that were taken from treated rats (Fig. 5C and D). Collagen deposits were found to surround the central veins, and none of these fibers appeared in the interlobular regions. Hepatic tissues from control and L-acetate-treated rats appeared to be similar when stained with reticulin stain (Fig. 5E and F).

According to an ANOVA analysis of variance, no significant changes were induced by the L-acetate fraction in the treated rats compared to the control non-treated rats in the concentrations of all the measured biochemical assays, namely, AST, ALT, cholesterol, HDL, protein, creatinine and glucose (Table 2).

### Chemical Constituents of the L-Acetate Fraction

**Figure 6:**
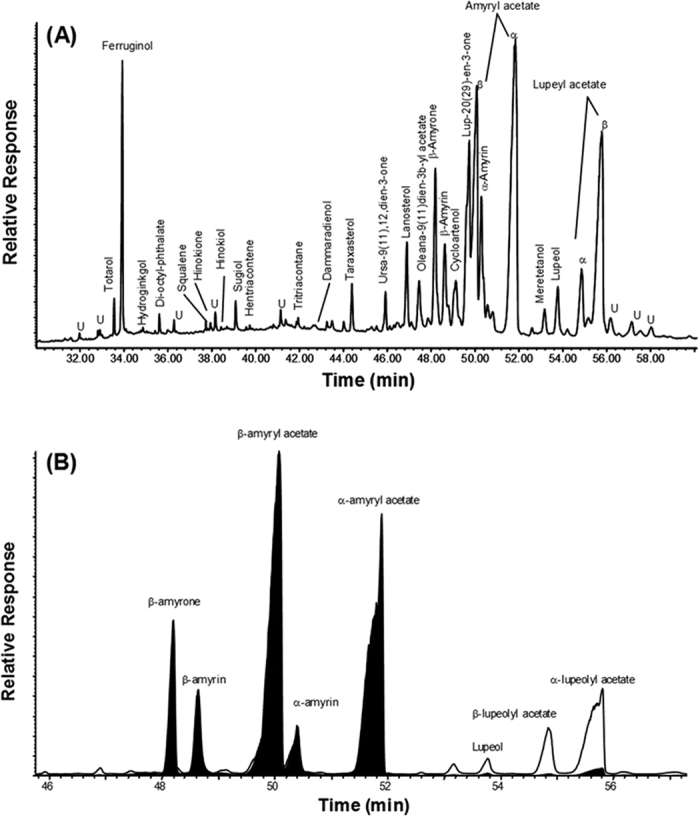
GC-MS analysis of L-Acetate fraction. (**A**) Total ion current (TIC) traces showing the major organic compounds. (**B**) Examples of typical GC-MS key ion plots (m/z 189/218) for triterpenoids (U = unknown).

The major L-acetate fraction’s relative concentrations of organic compounds are listed in Table 3, and the features of the GC-MS results for the propolis samples are shown in Fig. 6. The major extractable organic compound primarily included triterpenoids (74.0% of the total extract), steroids (9.8% of the total extract) and diterpenoids (7.9% of the total extract). The dominant compounds of the triterpenoids were α- and β-amyryl acetates (29.2% total extract), α- and β-lupeyl acetates (14.9% total extract), α- and β-amyrins (8.7% total extract), oleana-9(11)-dien-3β-yl acetate (2.4% total extract), lupeol (1.9% total extract), taraxasterol (1.4% total extract), urs-9(11),12-dien-3-one (1.3% total extract), and moretenol (1.1% total extract). The primary steroid compounds included cycloartenol (3.2% total extract), lanosterol (2.8% total extract), taraxasterol (1.4% total extract), lanostenyl acetate (1.3% total extract) and cycloartenyl acetate (1.1% total extract). The major diterpenoid compounds were primarily phenolics and included ferruginol (4.9% total extract), sugiol (1.3% total extract), totarol (1.0% total extract), hinokione (0.4% total extract), and hinokiol (0.3% total extract). No flavonoids were detected in the propolis from this region.

## Discussion

Many reports have described the biological activities of propolis samples that have been collected from different geographical regions of the world^25^,^51^,^77^,^78^,^79^,^80^. These properties include antimicrobial, antioxidant, anti-inflammatory, anti-viral and anticancer activities^69^,^72^,^79^,^81^,^82^,^83^,^84^. However, little is known about the biological activities of Saudi propolis, especially its effects on cancer cell lines. In this study, we investigated the anti-proliferative effect of an ethyl acetate fraction of local Saudi propolis (L-acetate fraction) against a number of cancer cell lines. In addition, we analyzed its chemical composition and its mechanism of action, and finally, we tested the side effects of this fraction in a rat model.

The major components of the L-acetate fraction were triterpenoids (74.0% of the total extract), steroids (9.8% of the total extract) and diterpenoids (7.9% of the total extract), which reflect the importance of Saudi propolis as a source of bio-active metabolites. Some of the components were detected as acetate salts and others were identified as phenols and alcohols. Triterpenoids are metabolites of isopentenyl pyrophosphate oligomers that are distributed throughout the plant kingdom^85^. The importance of triterpenoids as a source of medication for treating various chronic diseases is growing rapidly^86^. Many data suggest that both natural^87^,^88^ as well as synthetic triterpenoids^89^,^90^ have potential anticancer activities, and they act primarily by suppressing chronic inflammation by modulating pro-inflammatory mediators^86^. For example, α- and β-amyrin acetates that were isolated from *Alstonia boonei* are known to inhibit inflammation in animal models^91^. However, we could not find a direct or indirect relationship in the literature between these compounds and the anti-tubulin activity detected in this study.

Many di- and triterpenoid phenols and alcohols with known biological activities have also been detected in the L-acetate fraction. The most abundant diterpenoid phenol was ferruginol, with a relative abundance of 4.9%. Knowledge of its anti-tumor activity has already been published^92^. Additionally, ferruginol is known to induce apoptosis in a caspase-dependent manner in non-small lung cancer cells^93^. The second-most abundant diterpenoid phenol in the L-acetate fraction was sugiol, with a relative abundance of 1.3%. In addition to its anti-inflammatory^94^ and anticancer activities^95^, sugiol also exhibits antiviral activity against the H1N1 influenza virus *in vitro*^96^. The last detected diterpenoid phenol was totarol, with a relative abundance of 1% of the total fraction. Totarol is known to inhibit the growth of several gram-positive bacteria. Jaiswal *et al*.^97^ reported that totarol inhibits bacterial cytokinesis by interfering with the assembly and dynamics of Ftsz protein (a bacterial tubulin homolog)^97^,^98^. However, these researchers could not detect any effect on the HeLa cell microtubules^97^. Therefore, the anti-tubulin activity that has been detected in the L-acetate fraction seems to be induced via other components of the fraction and not from totarol. More recently, it has also been reported that totarol inhibits the secretion of some important *Staphylococcus aureus* virulence factors, namely the exotoxins alpha-hemolysin, staphylococcal enterotoxin A (SEA) and staphylococcal enterotoxin B (SEB)^99^. We also detected lupeol, a triterpenoid alcohol with antiangiogenic^100^ and anti-inflammatory^101^ activities. In addition to taraxasterol (1.4% abundance), a triterpenoid alcohol with anti-inflammatory and anti-arthritic activities was also found^102^.

All this information (when also considering the results of *in vivo* experiments) reflects the importance of Saudi propolis as a physiologically safe source of drug candidates with promising potential.

## Methods

### Propolis and metabolite extraction

The Al-Baha region of Saudi Arabia, which is located in the southern part of the country, was chosen as the location from which propolis was collected from stationed hives. The total area of the patio is 10,362 square km, and it is located in the southwestern part of Saudi Arabia (coordinates 41° 27′E/20° 0′N) with an altitude range from 1550 to 1900 meters, including mountainous areas up to 2215 m. The local bee colonies were classified as *Apis mellifera jemenitica.* The propolis sample was collected using a stainless-steel spatula and saved in Teflon-capped glass containers, which were labeled properly and stored in a freezer until the analysis and biological studies.

### Methanol crude extraction

Propolis (30 g) was extracted in methanol (300 ml, 95% v/v) by shaking the mixture at 150 rpm for 24 h at room temperature, and the suspension was then filtered. The residual propolis solid was further re-extracted and filtered in the same amount of methanol. The two extracts were pooled and kept at −20 °C for 24 h to precipitate the wax and resin. The mixture was then centrifuged and evaporated in a rotary evaporator (40 °C). The resulting residue (crude methanol extract) was weighed and dissolved in HPLC-grade methanol (Sigma Aldrich- Germany) at a final concentration of 10 mg/ml and stored at −20 °C until use.

### Column chromatography

Two grams of silica gel was packed into a glass column. Ten ml of crude methanol extract was then mixed with silica gel, evaporated to dryness and loaded on top of the silica gel-containing column. The column was then eluted with ethyl acetate (500 ml) followed by methanol (500 ml). Pressure was applied to speed the flow rate of the solvent through the column, and two fractions were collected. Each fraction was solvent-evaporated in a rotary evaporator (40 °C), and the residue was stored at −20 °C.

### Chemical Analysis

Chemical analysis by gas chromatography-mass spectrometry (GC-MS) was performed with a Hewlett-Packard 6890 gas chromatograph coupled to a 5973 Mass Selective Detector, using a DB-5MS (Agilent)-fused silica capillary column (30 m × 0.25 mm i.d., 0.25 μm film thickness) with helium as the carrier gas. The GC temperature was programmed to ramp from 65 °C (2 min initial hold) to 310 °C at 6 °C min^−1^ (it was isothermal for a 20-min final time), and the MS was operated in the electron impact mode at 70 eV of ion source energy. Mass spectrometric data were acquired and processed using the GC-MS ChemStation data system. The retention times were compared with those of the external standards. The compounds were identified by comparing them with the chromatographic retention characteristics and mass spectra of authentic standards, and the mass spectra and the mass spectral library of the GC-MS data system were reported. The mass spectra of unknown compounds were interpreted on the basis of their fragmentation patterns. The identification of triterpenoids, n-alkenes, n-alkanes and methyl n-alkanoates are based primarily on their mass spectra (i.e., the key ions at *m/z* values of 191/189/218, 97, 85, and 87, respectively). The compounds were quantified using the total ion current (TIC) peak area. A procedural blank was run in sequence with the propolis sample, and it presented no significant background interference.

### Cell proliferation assay

The growth inhibition was measured in a 96-well plate. Aliquots of 120 μl of the suspended cells (10^5^ ml^−1^) were added to 60 μl of serially diluted extracts in the cultivation media. After 4 days of incubation, the growth was determined by MTT assay as described previously^103^.

### Impedance Profiling

Impedance measurements of the treated and control cells were performed on an RT-CES system (xCELLigence) from Acea Biosciences (Roche) as described previously^104^. In brief, for the time-dependent cell response profiling, 60 μl of Dulbecco’s modified Eagle’s medium (DMEM) was added to 96-well E-Plates to obtain background readings, followed by the addition of 120 μl of L-929 cell suspension. The stock solution of the L-acetate fraction in DMSO was diluted with the cultivation medium to obtain a final test concentration for the IC_90_ of less than 0.1% DMSO. One μl of each prepared solution was then transferred into the 96-well E-Plate. Each E-plate also contained DMSO-only wells as the solvent control. The reference compounds and the ethyl acetate fraction with an unknown mode of action were measured in triplicate, and they were randomly distributed over the microtiter plates to avoid batch effects. The measurements were run for 5 days.

In using hierarchical cluster analysis and co-clustering, we compared the action of the reference compounds with a known mechanism of action to that of the L-acetate fraction to identify its mechanism of action.

### Cell cycle analysis

As described previously^105^, Jurkat cells were treated with different concentrations of the L-acetate fraction (or methanol (-ve control)) for 24 and 48 hours and then harvested by centrifugation. The cells were then fixed in methanol (80%) at −20 °C for 30 min, washed with PBS, and then treated with saponin (0.1% w/v) in PBS. Finally, the cells were treated with RNAse, and the nuclei were stained with propidium iodide (20 mgmL^−1^) for 30 min at 37 °C. The DNA content was measured with a FACSCalibur instrument (Becton Dickinson); 30,000 events were collected for each experiment. The data were analyzed with CellQuest software (Becton Dickinson).

### Microtubule staining

As described previously^105^, PtK_2_ cells were grown on glass cover slips in four-well plates. Exponentially growing cells were incubated with different concentrations of the extract for different time periods. The supernatant was removed and the cells were fixed in a −20 °C mixture of acetone/methanol (1 + 1) for 10 min. The fixed cells were washed twice with PBS and treated with anti-α-tubulin antibody (1:500 dilution, Sigma) for 45 min at 37 °C. The cells were then washed again with PBS and treated with anti-mouse-Alexa Flour 594 (1:10^4^ dilution, Molecular Probes). The nuclei were stained with DAPI (4-6-diamidino-2-phenylindole) solution (0.1 mg.ml^−1^) in PBS for 3 min. The cells were then examined with a fluorescence microscope equipped with the appropriate filters (Zeiss-Germany).

### Experimental animals

Male rats (*Rattus norvegicus*) were obtained from the college of pharmacy at King Saud University. The weight of each rat ranged from 150 to 170 grams. The animals were maintained at 18–22 °C in polypropylene cages and exposed to 12:12 h light:dark cycles. Before the experiment was started, the rats were allowed to acclimate to the laboratory environment for seven days. The study protocol was approved by the Animal Ethics Committee of the Zoology Department in the College of Science at King Saud University. For the experiment, the rats were divided into two groups (n = 10), namely a control group (receiving the vehicle) and a treated group (receiving a daily intra-peritoneal dose of 1 mg/kg body weight of the L-acetate fraction for ten days).

### Blood, kidney and liver samples

The animals were autopsied under light ether anesthesia. The blood needed for the experiment was obtained from the retro-orbital venous sinus. The liver enzymes, total protein and lipid profiles were estimated from the separated plasma. The kidney and liver were washed in cold saline and processed for histological studies.

### Liver function tests and lipid profile

The concentrations of liver enzymes, HDL, cholesterol, protein, creatinine and glucose in the serum were estimated according to the manufacturer’s protocols (Bio Merieux kits, France). A UV/Visible-Model-80-2106-00 spectrophotometer (Pharmacia Biotech, Cambridge, England) was used to determine the colorimetric responses of the proteins.

### Histological study

The kidney and liver tissue sections were processed using light microscopy. The sections were fixed in 10% neutral formalin, embedded in paraffin and then stained with hematoxylin-eosin, Masson trichrome and reticulin stains. The tissue damage was investigated in a blind fashion using a DMRB/E light microscope (Leica, Heerbrugg, Switzerland).

### Statistical analysis

MINITAB software (MINITAB, State College, PA, Version 13.1, 2002) was used to analyze the data. The data were tested for normality (with the Anderson Darling test and for variance homogeneity) prior to any further statistical analysis. The normally distributed data with homogeneous variances were analyzed using a one-way ANOVA. The results were expressed as the means (M) ± standard deviation (SD).

### Ethical Clearance

All animal procedures were conducted in accordance with the standards set forth in the guidelines for the care and use of experimental animals by the Committee for the Purpose of Control and Supervision of Experiments on Animals (CPCSEA) and the National Institutes of Health (NIH). The study protocol was approved by the Animal Ethics Committee of the Zoology Department in the College of Science at King Saud University, KSA.

## Conclusions

The L-acetate bio-active fraction of Saudi propolis that was isolated from the Al-Bahah region has been characterized using GC-MS techniques. The major compounds were triterpenoids and diterpenoids. The predominant triterpenoids were α- and β-amyryl acetates, α- and β-amyrins, and α- and β-lupeyl acetates, followed by lupeol, ferruginol, sugiol, taraxasterol and totarol. The fraction showed anti-tubulin activity combined with G_2_M cell cycle arrest and apoptotic induction in cancer cell lines. This study reinforces the need to understand the therapeutic potential of Saudi propolis.

## Additional Information

**How to cite this article**: Elnakady, Y. A. *et al*. Characteristics, chemical compositions and biological activities of propolis from Al-Bahah, Saudi Arabia. *Sci. Rep.* **7**, 41453; doi: 10.1038/srep41453 (2017).

**Publisher's note:** Springer Nature remains neutral with regard to jurisdictional claims in published maps and institutional affiliations.

## Figures and Tables

**Table 1:** IC_50_-Values for a crude methanol extract of propolis and the L-acetate fraction in four different cell lines as measured by MTT Assay.

Cell line	ATCC-	Disease	IC_50_-Methanol Extract (μg/ml)	IC_50_-L-acetate fraction (μg/ml)
Jurkat	TIB152	Acute T leukemia	**70**	**3.2**
A549	CRM-CCL-185	Lung carcinoma	**37**	**1.8**
HepG2	HB-8065	Hepatocellular carcinoma	**3**	**6.3**
SW756	CRL-10302	Squamous carcinoma	**200**	**2.8**

**Table 2:** Serum concentrations of AST, ALT, cholesterol, HDL, protein, creatinine and glucose in control and treated rats.

	Control group	Treated group
ALT (U/L)	236 (±8.4)	271 (±12)
AST (U/L)	52 (±1.9)	64 (±2.3)
Cholesterol (mg/Dl)	55 (±2.8)	67 ± (2.6)
HDL-Cholesterol (mg/Dl)	40 (±1.4)	35 (±2)
Creatinine (mg/Dl)	2.1 (±0.6)	2.2 (±0.7)
Protein (g/100 ml)	6.5 (±0.2)	6.1 (±0.15)
Glucose (mg/Dl)	131 (±5.8)	125 (±6.2)

**Table 3:** Relative concentrations (%) of the major compounds in the L-acetate fraction of propolis from the Al-Baha region in Saudi Arabia.

Compound	Composition	MW	Relative Concentration (%)
Totarol	C_20_H_30_O	286	1.0
Ferruginol	C_20_H_30_O	286	4.9
Methyl octadecenoate	C_19_H_36_	296	0.07
Hinokione	C_20_H_28_O_2_	300	0.4
Sugiol	C_20_H_28_O_2_	300	1.3
Hinokiol	C_20_H_30_O_2_	302	0.31
Hydroginkgol	C_21_H_36_O	304	0.84
Squalene	C_30_H_50_	410	0.7
Urs-9(11), 12 dien-3-one	C_30_H_46_O	422	1.3
Dammaradienol	C_30_H_50_O	426	1.07
Taraxasterol	C_30_H_50_O	426	1.4
Lup-20(29)en-3-one	C_30_H_48_O	424	8.1
α-amyrin	C_30_H_48_O	424	4.8
Lanosterol	C_30_H_50_O	426	2.8
β-amyrone	C_30_H_50_O	426	5.1
β-amyrin	C_30_H_48_O	424	3.9
Cycloartenol	C_30_H_50_O	426	3.2
Moretenol	C_30_H_50_O	426	1.1
Lupeol	C_30_H_50_O	426	1.9
Olean-13(18)-en-3-one	C_30_H_50_O_2_	426	0.25
Hentriacontene	C_31_H_62_	434	0.67
Tritriacontene	C_33_H_68_	462	0.89
Oleana-9(11)-dien-3β-yl acetate	C_32_H_50_O_2_	466	2.4
β-amyryl acetate	C_32_H_52_O_2_	468	11
Cycloartenyl acetate	C_32_H_52_O_2_	468	1.1
Lanostenyl acetate	C_32_H_52_O_2_	468	1.3
α-amyryl acetate	C_32_H_52_O_2_	468	18.2
β-lupenyl acetate	C_32_H_52_O_2_	468	2.8
α-lupenyl acetate	C_32_H_52_O_2_	468	12.1
Di-octyl-phthalate	C_24_H_38_O_4_	390	0.6
**Total**			95.5
